# Effect of Cancer Stage on Adverse Kidney Outcomes in Patients Receiving Immune Checkpoint Inhibitors for Melanoma

**DOI:** 10.1016/j.ekir.2022.08.030

**Published:** 2022-09-08

**Authors:** Qiyu Wang, Ian A. Strohbehn, Sophia Zhao, Harish Seethapathy, Samuel D. Strohbehn, Paul Hanna, Meghan Lee, Riley Fadden, Ryan J. Sullivan, Genevieve M. Boland, Kerry L. Reynolds, Meghan E. Sise

**Affiliations:** 1 Department of Medicine, Division of Nephrology, Massachusetts General Hospital, Boston, Massachusetts, USA; 2 Department of Medicine, Division of Hematology and Oncology, Massachusetts General Hospital, Boston, Massachusetts, USA; 3 Division of Surgery, Massachusetts General Hospital, Boston, Massachusetts, USA

**Keywords:** anticytotoxic T-lymphocyte-associated protein 4, antiprogrammed cell death protein 1, chronic kidney disease, immune checkpoint inhibitor-associated AKI, immune-related adverse events, melanoma

## Introduction

Immune checkpoint inhibitors (ICIs) have transformed the treatment of melanoma. Since the approval of ipilimumab, a humanized cytotoxic T-lymphocyte associated antigen 4 antibody (anti-CTLA-4) in 2011, pembrolizumab and nivolumab (both targeting programmed cell death protein 1 [anti-PD-1]), as well as combination therapy (ipilimumab/nivolumab) have been shown to prolong progression-free survival and were approved for treatment of stage 4 melanoma. ^1^^,^S1,S2 Most recently, ICIs have been approved as adjuvant therapy for surgically resected stage 3 melanoma. S3,S4 Nevertheless, their success in cancer response comes with the cost of immune-related adverse events. ICI-associated acute kidney injury (AKI), characterized by acute tubulointerstitial nephritis on pathology, was estimated to have an incidence of 2% to 5%.^2^, ^3^, ^4^^,^S6^,^S5 Differentiating between ICI-associated AKI and other causes of AKI is important.^2^, ^3^, ^4^^,^S7–S12

To better understand the effect of cancer stage on adverse kidney outcomes, we retrospectively evaluated the incidence and etiology of AKI and long-term kidney function decline in a large cohort of patients with stage 3 and stage 4 melanoma treated with ICIs.

## Results

Among the 848 ICI-treated patients with advanced melanoma included in the study, 640 patients were treated with anti-PD-1 monotherapy (*N* = 251 stage 3, *N* = 389 stage 4) and 208 patients were treated with combination anti-CTLA-4/PD-1 therapy (all stage 4) (Supplementary Figure S1). The average age was 63.6 years (SD 13.8), 60% were men, and 97% were White. The mean baseline estimated glomerular filtration rate was 87 ml/min per 1.73 m^2^. The distributions of sex, race, and comorbidities were similar across the 3 groups (Supplementary Table S1).

### Incidence of All-cause AKI and Comparison of Etiologies

**Figure 1:**
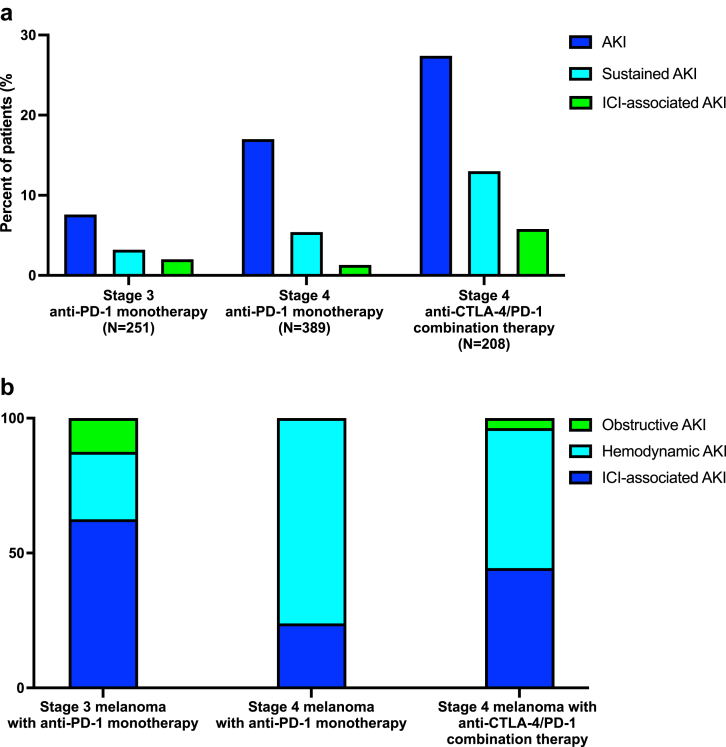
Comparison of incidence and etiology of AKI by cancer stage and immune checkpoint inhibitor (ICI) regimen (a) Comparison of incidence of AKI, sustained AKI, and ICI-associated AKI between patients with stage 3 melanoma treated with anti-PD-1 monotherapy, stage 4 melanoma treated with anti-PD-1 monotherapy, and stage 4 melanoma treated with anti-CTLA-4/PD-1 monotherapy. AKI was defined by ≥1.5-fold increase in creatinine from pre-ICI baseline; sustained AKI was a subset of all AKI cases that lasted for >48 hours. ICI-associated AKI was diagnosed by either kidney biopsy or clinical criteria (adjudicated by 2 nephrologists from chart review of all cases of sustained AKI, see method section for further details). (b) Comparison of AKI etiologies by cancer stage and ICI regimen among all patients with AKI sustained >48 hours. Hemodynamic AKI includes prerenal azotemia, ischemic or septic acute tubular necrosis, and toxic acute tubular necrosis (nephrotoxic medications/contrast-induced tubular injury, pigment nephropathy, tumor lysis syndrome). ICI-associated AKI was diagnosed by either kidney biopsy or clinical criteria (adjudicated by 2 nephrologists from chart review of all cases of sustained AKI, see method section for further details). Obstructive AKI occurred with documented ureteral or urinary outlet obstruction. If more than 1 episodes of sustained AKI with different etiologies occurred in an individual patient, ICI-associated AKI was chosen as the dominant outcome, followed by obstructive AKI, then hemodynamic AKI. AKI, acute kidney injury; anti-CTLA-4, anticytotoxic T-lymphocyte associated protein 4; anti-PD-1, antiprogrammed cell death protein 1.

The incidence of all-cause AKI was 16.7%, sustained AKI 6.6%, and ICI-associated AKI 2.6% within 1-year follow-up of the entire cohort. AKI rates were significantly higher among patients with stage 4 melanoma (27.4% in patients treated with anti-CTLA-4/PD-1 combination therapy, 17.0% in patients treated with anti-PD-1 monotherapy), compared to 7.6% in patients with stage 3 melanoma receiving anti-PD-1 monotherapy (Figure 1a, Supplementary Figure S1).

After clinical adjudication, we determined that the rate of ICI-associated AKI was similar between stage 4 and stage 3 patients treated with anti-PD-1 monotherapy (1.3% vs. 2.0%, *P* = 0.49), and was significantly higher among patients with stage 4 melanoma treated with anti-CTLA-4/PD-1 combination therapy (5.8%, *P* < 0.01) (Figure 1a). Hemodynamic AKI was much more common among patients with stage 4 melanoma, whereas among patients with stage 3 melanoma, ICI-associated AKI was the more common cause of sustained AKI (62.5%) (Figure 1b).

### Predictors of All-cause AKI and ICI-associated AKI

**Figure 2:**
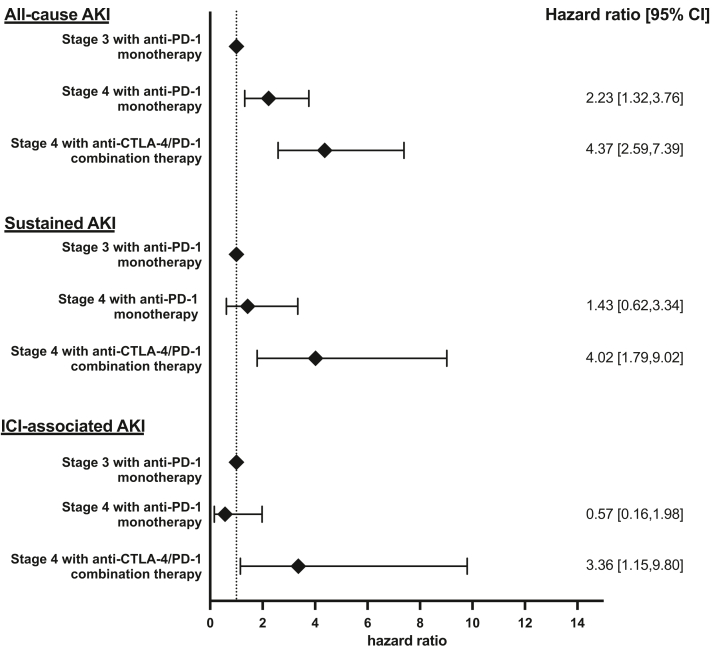
Forest plot of hazard ratios for AKI, sustained AKI and immune checkpoint inhibitors (ICI)-associated AKI by melanoma stage and ICI treatment regimen. Forest plot showing the subdistrubution hazard ratios (sHR) for AKI, sustained AKI and ICI-associated AKI by melanoma stage and ICI treatment regimen. sHR for AKI, sustained AKI, and ICI-associated AKI in patients with stage 4 melanoma treated with anti-PD-1 monotherapy and stage 4 melanoma treated with anti-CTLA-4/PD-1 combination therapy (using stage 3 melanoma as the reference group) from multivariable Fine-Gray models were shown. For the outcome of all-cause AKI, the model was adjusted for age, sex, race, group, diabetes, hypertension, and use of PPI. For the outcome of sustained AKI, the model was adjusted for age, sex, race, group, baseline estimated glomerular filtration rate, and use of proton pump inhibitors. For the outcome of ICI-associated AKI, the model was adjusted for age, sex, race and group. AKI, acute kidney injury; anti-CTLA-4, anticytotoxic T-lymphocyte associated protein 4; anti-PD-1, antiprogrammed cell death protein 1.

In the multivariable Fine-Gray model, stage 4 melanoma treated with anti-PD-1 monotherapy was associated with more than 2-fold risk of developing all-cause AKI (estimated subdistribution hazards ratios [sHR], 2.23; 95% confidence interval [CI], 1.32–3.76, *P* < 0.01), whereas stage 4 melanoma treated with anti-CTLA-4/PD-1 combination therapy was associated with more than 4-fold risk (sHR 4.37; 95% CI, 2.59–7.39, *P* < 0.01) compared to patients with stage 3 melanoma (Figure 2).

For ICI-associated AKI, only anti-CTLA-4/PD-1 combination therapy use was significantly associated with the outcome (sHR 3.36; 95% CI, 1.15–9.80, *P* = 0.03) (Figure 2, Supplementary Table S2). There was no increase in the risk of ICI-associated AKI in patients with stage 4 melanoma receiving anti-PD-1 monotherapy compared to stage 3 melanoma. Case summaries of all cases of sustained AKI are shown in Supplementary Table S3.

### Chronic Kidney Function Decline Among Survivors

A total of 631 patients (74.1%) were alive at 1 year follow-up (Supplementary Table S4). Patients with stage 4 melanoma had higher 1-year mortality rates compared to patients with stage 3 melanoma (32.3% vs. 9.6%, *P* < 0.01). There were no significant differences in the cumulative incidence of composite chronic kidney disease (CKD) outcome based on cancer stage or ICI regimen (Supplementary Figure S2). Among patients who survived 4 years, 15.4% developed composite CKD outcome. A multivariable Fine-Gray model found that advanced age (>65 years old, sHR 4.61; 95% CI, 2.10–10.09 *P* < 0.01) and lower baseline estimated glomerular filtration rate (sHR 1.14 for every 10 ml/min per 1.73 m^2^ decrease in baseline estimated glomerular filtration rate; 95% CI, 1.02, 1.28, *P* = 0.02) were both significant risk factors for composite CKD outcome (Supplementary Table S5).

### AKI and Risk of Mortality

Patients who developed non-ICI-associated AKI within 6 months after ICI initiation had a significantly increased risk of mortality compared to those who were alive and did not have AKI within 6 months (adjusted hazard ratio, aHR 1.46; 95% CI, 1.08–1.97, *P* = 0.01); ICI-associated AKI within 6 months was not associated with increased risk of mortality (aHR 0.84; 95% CI, 0.34–2.05, *P* = 0.70) (Supplementary Table S6).

## Discussion

In this large, single health care network, retrospective cohort study evaluating the incidence of all-cause AKI, ICI-associated AKI, and CKD in patients with advanced melanoma treated with ICIs, we found that all-cause AKI incidence was substantially higher among patients with stage 4 melanoma compared to those stage 3 melanoma, whereas the risk of ICI-associated AKI was similar between stage 3 and stage 4 patients treated with anti-PD-1 therapy, and only higher among patients with stage 4 melanoma who received anti-CTLA-4/PD-1 combination therapy. ICI-associated AKI was the most common cause of sustained AKI in patients with stage 3 melanoma, whereas hemodynamic AKI was the most common cause in patients with stage 4 melanoma. The high incidence of hemodynamic AKI in metastatic melanoma likely reflects the vulnerability of such patients to hemodynamic insults, infection, and high risk of nephrotoxic medication exposure. We also found that the composite CKD outcome increased significantly as survival increases, with equivalent risks among survivors with stage 3 or stage 4 melanoma. This has important clinical implications because ICIs are now approved as adjuvant therapy for numerous types of cancers (melanoma, renal cell carcinoma, esophageal, lung, and breast cancer) and the number of cancer survivors treated with ICIs are expected to grow exponentially; S13–S16 thus, careful long-term monitoring of kidney function is warranted.

Finally, we detected an increase in mortality among patients who developed hemodynamic or obstructive AKIs (non-ICI-associated AKI) within the first 6 months. Previous studies have suggested that patients with immune-related adverse events, including ICI-associated AKI, may have improved survival because it might represent therapeutic response to ICIs;S12 we did not find a statistically significant association between ICI-associated AKI and mortality in our study.

Our study has several limitations. First, retrospectively defining AKI could potentially lead to underestimation of the incidence of AKI if an AKI event happened outside our healthcare network. Second, because accurate estimation of kidney function is challenging in patients with cancer who commonly have sarcopenia; creatinine-based estimated glomerular filtration rate equation may provide a less accurate estimation of the true kidney function, and can lead to underestimation of AKI or CKD events.S17,S18 Third, over the counter medication use, including nonsteroidal anti-inflammatory drugs or proton pump inhibitors, may not be captured by the electronic medical record, making adjustment of these confounding factors challenging. In addition, we did not stratify patients with stage 3 melanoma by their substages, and a small subset of stage 3 patients may have had surgically unresectable disease, which carries worse prognosis compared to surgically resected disease.S19 Finally, only 3 out of the 22 ICI-associated AKI cases (13.6%) underwent biopsy, similar to the average rate of kidney biopsy (7%∼30%) in cohort studies that examined the risk of AKI after ICIs.^4^^,^S8,S10 Though the updated guidelines recommended that kidney biopsy should be strongly considered in diagnosing ICI-associated AKI,S20 concerns about the peri-procedural bleeding risk of kidney biopsy, especially in patients with malignancy who may require antiplatelet or anticoagulant therapy, has posed significant challenge to obtaining kidney biopsies in real life practice.^5^ Prior studies have suggested that immune-related adverse events is an important predictor of ICI-associated AKI, however, we found that numerous patients with concurrent immune-related adverse events had other causes of AKI, highlighting the diagnostic challenges in this disease (Supplementary Table S3). Clinicians have proposed and utilized clinical diagnostic criteria for ICI-associated AKI based on clinical features and risk factors obtained from rigorously conducted clinical studies.^6^, ^7^, ^8^ Our study highlights that the pretest probability of ICI-associated AKI differs by cancer stages and ICI regimen.

In this real-world study of ICI use in the era of adjuvant therapy for melanoma, we found that risk of all-cause AKI increases with more advanced cancer stage, yet the risk of ICI-associated AKI is only driven by the use of anti-CTLA-4/PD-1 combination therapy and not cancer stage. Despite a lower absolute risk of all-cause AKI, ICI-associated AKI is the most common cause of sustained AKI in a patient with stage 3 melanoma. We also found equivalent risk of composite CKD outcome among survivors regardless of cancer stage or ICI regimen used, which implicates the importance of long-term kidney function monitoring, especially in patients with stage 3 melanoma treated with anti-PD-1 adjuvant therapy who are expected to be long-term survivors.

## Disclosure

MES has served on a scientific advisory board panel for Mallinckrodt. RJS serves as a consultant and/or on scientific advisory boards for BMS, Merck, Novartis, and Pfizer; and his institution has received grant funding from Merck to support his research. KR received grant funding from Project Datasphere. GMB has sponsored research agreements with Olink Proteomics, InterVenn Biosciences, and Palleon Pharmaceuticals. She has been on scientific advisory boards for Merck, Novartis, Nektar Therapeutics, and Iovance. She has been a consultant for Merck, Ankyra Therapeutics, and InterVenn Biosciences. RF has been on scientific advisory board for Apricity Health. QW, SZ, HS, PH, IS, SDS and ML do not have conflict of interest to declare.

## Supplementary Material

Supplementary File (PDF)

**Supplement** **ary Methods.**

**Supplementary References.**

**Figure S1.** Patient flow

**Figure 2.** Cumulative incidence curve for composite chronic kidney disease outcome by melanoma stage and ICI treatment regimen among patients surviving more than 1 year

**Table S1.** Baseline characteristics of patients by melanoma stage and immune checkpoint inhibitor regimen

**Table S2.** Univariable and multivariable Fine-Gray model predicting development of all-cause AKI and ICI-associated AKI within 1-year after ICI initiation with death as a competing risk

**Table S3.** Case summaries of 56 patients who developed at least 1 episode of sustained AKI within 1-year of ICI initiation

**Table S4.** Baseline characteristics of patients who survived more than 1 year by melanoma stage and ICI treatment regimen

**Table S5.** Univariable and multivariable Fine-Gray model predicting composite CKD outcome among patients surviving more than 1 year

**Table S6.** Univariable and multivariable Cox model evaluating association of AKI with mortality in patients survived more than 6 months
