# Supplementary material for: Effect of Cancer Stage on Adverse Kidney Outcomes in Patients Receiving Immune Checkpoint Inhibitors for Melanoma
Source: Kidney Int Rep. 2022 Sep 8;7(11):2517–21. doi: 10.1016/j.ekir.2022.08.030 (PMC9751690; doi:10.1016/j.ekir.2022.08.030)
Supplement: Supplementary File (PDF) [file mmc1.pdf]

Table of Contents

Supplementary Methods

Supplementary Tables---2-4
Table S1. Baseline characteristics of patients by melanoma stage and immune checkpoint inhibitor regimen--5
Table S2. Univariable and multivariable Fine-Gray model predicting development of all-cause AKI and ICI-associated AKI within 1-year after ICI initiation with death as a competing risk---6
Table S3: Case summaries of 56 patients who developed at least 1 episode of sustained AKI within 1-year of ICI initiation------------------7-10
Table S4. Univariable and multivariable Fine-Gray model predicting composite CKD outcome among patients surviving ≥1 year------------11
Table S5. Baseline characteristics of patients who survived more than one year by melanoma stage and ICI treatment regimen--------------12
Table S6. Univariable and multivariable Cox model evaluating association of AKI with mortality in patients survived ≥6 months------------13

Supplementary Figures
Figure S1. Patient Flow--14
Figure S2: Cumulative incidence curve for composite chronic kidney disease outcome by melanoma stage and ICI treatment regimen among patients surviving more than one year--15

Supplementary References---16

Supplementary Methods

Study design and patient population

We conducted a retrospective cohort study including all patients diagnosed with advanced melanoma who initiated ICIs between January 2016 and December 2019 at Mass General Brigham. Exclusion criteria included lack of baseline creatinine within 30 days prior to ICI initiation, lack of follow-up creatinine within 1 year after ICI initiation, or end-stage kidney disease prior to ICI initiation. The follow-up period began on the date of first ICI exposure and continued until December 31, 2020, 1 year after the last date of enrollment. Patients were grouped into 3 cohorts: Stage 3 melanoma (all received anti-PD-1 monotherapy), Stage 4 melanoma treated with anti-PD-1 monotherapy, and Stage 4 melanoma treated with anti-CTLA-4/PD-1 combination therapy. The decision regarding ICI regimen used in Stage 4 disease was made by the treating oncologist and was determined by metastatic burden, central nervous system involvement, and patient frailty and preference. This protocol was approved by the Mass General Brigham IRB and the need for informed consent was waived.

Data collection

Date of ICI initiation and ICI type (nivolumab, pembrolizumab and combination ipilimumab/nivolumab) were obtained from oncology infusion records. Melanoma disease stage was extracted by chart review of the oncology clinic note on the date of ICI initiation. Covariates including age, sex, race, comorbidities, and medications were obtained from Research Patient Data Repository at Mass General Brigham. Baseline creatinine was defined as the value closest to the date of ICI initiation and estimated glomerular filtration rate (eGFR) was calculated using the CKD-EPI 2021 equation. [S26]

Study definitions

AKI was defined as a ≥1.5-fold increase in creatinine from baseline within 12 months of ICI initiation. Sustained AKI was defined as AKI that lasted for >48 hours, and all episodes of sustained AKI were chart reviewed by two nephrologists, and a third nephrologist resolved disagreement in adjudications. The cause of AKI was divided into three categories: hemodynamic AKI, ICI-associated AKI, and obstructive AKI. Hemodynamic AKI included pre-renal azotemia, ischemic/septic acute tubular necrosis (ATN), and toxic ATN (nephrotoxic medications/contrast-induced tubular injury, pigment nephropathy, tumor lysis syndrome). ICI-associated AKI was diagnosed by either kidney biopsy (acute tubulointerstitial inflammation on pathology) or clinical criteria: sustained AKI episode directly attributed to the ICIs by the adjudicators meeting at least 2 of the following 3 criteria: 1) ICIs held for at least 1 cycle due to concern for ICI-associated acute tubulointerstitial nephritis (ATIN), or 2) treatment with steroids (>0.5mg/kg) due to concern for ICI-associated ATIN, or 3) concurrent irAE is present (within 2 weeks of AKI development) without a plausible alternative cause of AKI. Obstructive AKI was defined by AKI that developed due to ureteral or urinary outlet obstruction. Non-ICI-associated AKIs include sustained AKI episodes that were attributed to non ICI-related causes (hemodynamic or obstructive AKIs) and those AKI episodes lasted less than 48 hours (presumed to have hemodynamic causes of AKI given rapid improvement).

Primary and secondary outcomes

The co-primary outcomes were the incidence of all-cause AKI and ICI-associated AKI within 1-year of ICI initiation among all patients starting ICIs. In the subset of patients surviving more than one year, we examined two additional outcomes to determine the risk of chronic kidney function decline after ICIs. The CKD outcome was the composite outcome of a new onset CKD (defined as eGFR < 60 mL/min/1.73m^2 at least twice separated by at least 90 days without intervening values above 60 mL/min/1.73m^2) or experiencing a sustained 30% decline in eGFR compared to baseline for >90 days, whichever happened first. We limited the analysis of CKD only to patients surviving at least one year because the chronic sequelae of ICIs are not as clinically meaningful in patients whose life expectancy is significantly limited by malignancy. Death was determined from

electronic medical records or was assigned 45 days after the last day of laboratory result in patients witout datapoints for more than 9 months. Except death, the follow-up for each outcome was censored at the time of the first occurrence of the outcome of interest, or at the end of study (December 31, 2020).

Statistical analysis

Baseline characteristics were described using means and standard deviations (SD) for continuous variables and counts and percentages for categorical variables. The primary outcomes of all-cause AKI and ICI-associated AKI were evaluated by competing risks analysis using the Fine-Gray subdistribution hazard model. Age, sex, race, ICI treatment group (Stage 3 anti-PD-1 monotherapy, Stage 4 anti-PD-1 monotherapy, and Stage 4 anti-CTLA-4/PD-1 combination therapy), and other baseline characteristics with a P value < 0.1 in the univariate analysis were included in the multivatiate model. A final parsimonious model was developed based on clinical plausibility. Similar analyses were also performed for the composite outcome of CKD. The COX proportional-hazards regression models were performed to evaluate the association between AKI occurred within 6 months of ICI initiation (ICI-associated AKI and non-ICI-associated AKI, respectively) and the risk of mortality compared to patients without AKI within 6 months. All statistical tests were 2-sided, and $P < 0.05$ was considered statistically significant. The analyses were performed with Stata/SE 17.0.

Supplementary Table 1. Baseline characteristics of patients by melanoma stage and immune checkpoint inhibitor regimen

Characteristics	All patients (N=848)	Stage 3 melanoma (N=251)	Stage 4 melanoma treated with anti-PD-1 monotherapy (N=389)	Stage 4 melanoma treated with anti-CTLA-4/PD-1 combination therapy (N=208)
**Demographics**				
**Age (years, SD)**	63.6 (13.8)	62.5 (14.5)	67.1 (12.9)	58.3 (12.6)
**male sex (N, %)**	507 (59.8%)	143 (57.0%)	245 (63.0%)	119 (57.2%)
**Race (N, %)**				
**White**	820 (96.7%)	239 (95.2%)	378 (97.2%)	203 (97.6%)
**Other**[1]	28 (3.3%)	12 (4.8%)	11 (2.8%)	5 (2.4%)
**Co-existing conditions-no.(%)**				
**Hypertension**	492 (58.0%)	142 (56.6%)	244 (62.7%)	106 (51.0%)
**Diabetes**	152 (17.9%)	42 (16.7%)	76 (19.5%)	34 (16.3%)
**Cirrhosis**	5 (0.6%)	0 (0%)	3 (0.8%)	2 (1.0%)
**CAD**	156 (18.4%)	46 (18.3%)	83 (21.3%)	27 (13.0%)
**eGFR at ICI initiation (ml/min, SD)**	86.8 (19.3)	86.4 (19.4)	84.1 (19.6)	92.4 (17.4)
**CKD stage at ICI initiation**				
**eGFR ⩾90 ml/min/1.73m^2**	411 (48.5%)	122 (48.6%)	160 (41.1%)	129 (62.0%)
**60-89 ml/min/1.73m^2**	357 (42.1%)	105 (41.8%)	183 (47.0%)	69 (33.2%)
**45-59 ml/min/1.73m^2**	54 (6.4%)	16 (6.4%)	33 (8.5%)	5 (2.4%)
**30-44 ml/min/1.73m^2**	23 (2.7%)	7 (2.8%)	11 (2.8%)	5 (2.4%)
**<30 ml/min/1.73m^2**	3 (0.4%)	1 (0.4%)	2 (0.5%)	0
**Medication use-no.(%)**				
**ACEI/ARB**	287 (33.8%)	77 (30.7%)	150 (38.6%)	60 (28.8%)
**PPI**	296 (34.9%)	66 (26.3%)	160 (41.1%)	70 (33.7%)
**Diuretics**[2]	246 (29.0%)	56 (22.3%)	141 (36.2%)	49 (23.6%)
**ICI agent-no.(%)**				
**Pembrolizumab**	433 (51.1%)	134 (53.4%)	299 (76.9%)	-
**Nivolumab**	207 (24.4%)	117 (46.6%)	90 (23.1%)	-
**Combination ipilimumab/nivolumab**	208 (24.5%)	-	-	208 (100%)

Table S1. Abbreviations: ICI = immune checkpoint inhibitor, CAD = coronary artery disease, eGFR = estimated glomerular filtration rate, ACEI/ARB = angiotensin converting enzyme inhibitor/angiotensin receptor blockade, PPI = proton pump inhibitor, anti-CTLA-4 = anti-cytotoxic T-lymphocyte associated protein 4, anti-PD1 = anti-programmed cell death protein 1. 1) Includes Asian, Hispanic, African American (total of 8 patients), and those races that were not documented (total of 20 patients). 2) Includes loop, thiazide-like, and potassium-sparing diuretics

Supplementary Table 2. Univariable and multivariable Fine-Gray model predicting development of all-cause AKI and ICI-associated AKI within 1-year after ICI initiation with death as a competing risk

	All-cause AKI				ICI-associated AKI			
**Characteristics**	**Univariable analysis**	**P-value**	**Multivariable analysis**	**P-value**	**Univariable analysis**	**P-value**	**Multivariable analysis**	**P-value**
	Hazard ratio [95% CI]		Hazard ratio [95% CI]		Hazard ratio [95% CI]		Hazard ratio [95% CI]	
**Age**	1.00 [0.99, 1.02]	0.53	1.00 [0.99,1.02]	0.44	1.01 [0.98,1.03]	0.58	1.02 [0.99,1.05]	0.12
**Female sex**	1.08 [0.78, 1.51]	0.65	1.14 [0.80,1.62]	0.47	0.43 [0.16,1.18]	0.10	0.42 [0.16,1.08]	0.07
**White race**	0.77 [0.35, 1.71]	0.53	0.60 [0.27,1.34]	0.21	0.72 [0.10,5.27]	0.74	0.44 [0.06,3.18]	0.40
**Stage 3 melanoma**	1		1		1		1	
**Stage 4 melanoma with anti-PD-1 monotherapy**	2.39 [1.44,3.95]	0.001	2.23 [1.32,3.76]	0.003	0.64 [0.19,2.22]	0.48	0.57 [0.16,1.98]	0.38
**Stage 4 melanoma with anti-CTLA-4/PD-1 combination therapy**	4.19 [2.51,7.01]	<0.001	4.37 [2.59,7.39]	<0.001	2.98 [1.05, 8.43]	0.04	3.36 [1.15,9.80]	0.03
**Every 10ml/min/1.73m^2 decrease in baseline eGFR**	0.93 [0.85, 1.02]	0.13			0.93 [0.80, 1.10]	0.41		
**Diabetes**	1.43 [0.97, 2.11]	0.07	1.27 [0.86, 2.01]	0.21	0.72 [0.21,2.43]	0.60		
**Cirrhosis**	2.83 [0.69, 11.55]	0.14			0			
**HTN**	1.38 [0.98, 1.94]	0.07	1.30 [0.89, 1.90]	0.18	0.50 [0.21, 1.17]	0.11		
**CAD**	1.06 [0.69, 1.61]	0.79			0.44 [0.10, 1.87]	0.27		
**PPI**	1.51 [1.08, 2.10]	0.02	1.30 [0.90, 1.88]	0.16	1.56 [0.67, 3.60]	0.30		
**ACEi/ARB***	1.40 [1.00, 1.95]	0.05						
**Diuretics***	1.47 [1.04, 2.07]	0.02						

Table S2. In both multivariable models, death was a competing risk. Baseline demographics (age, race, sex) and group were selected a priori for inclusion. Additionally, baseline variables with a *P* value<0.1 in the univariable model (diabetes, HTN, PPI use) were also included. *ACEi and diuretics use were not included in the multivariate model given their correlation with diabetes and HTN. Stage 3 melanoma were used as the reference group as it is associated with lowest incidence of AKI. AKI was defined as ≥1.5-fold increase in creatinine from baseline within 12 months of ICI initiation. ICI-associated AKI was diagnosed either by kidney biopsy or clinical criteria (see method section for further details). Abbreviations: anti-CTLA-4 = anti-cytotoxic T-lymphocyte-associated protein 4, anti-PD-1= anti-programmed cell death protein 1, eGFR = estimated glomerular filtration rate, HTN = hypertension, CAD = coronary artery disease, ACEI/ARB = angiotensin converting enzyme inhibitor/angiotensin receptor blockade, PPI = proton pump inhibitor. Diuretics includes loop, thiazide-like, and potassium-sparing diuretics

Supplementary Table 3: Case summaries of 56 patients who developed at least 1 episode of sustained AKI within 1-year of ICI initiation

ID	Age, Race, Sex	Disease stage	ICI regimen	Baseline creatinine (mg/dl)	Peak creatinine (mg/dl)	Time to AKI (days)	Concurrent irAE	Case summary
**1**	85 yo WM	Stage 3	pembrolizumab	1.06	3.02	9	Pyrexia	Clinically adjudicated ICI-associated AKI improved with steroids and holding ICI
**2**	50 yo WM	Stage 3	nivolumab	1.02	3.03	294		Biopsy-confirmed severe AIN improved with steroids and holding therapy
**3**	55 yo WM	Stage 3	nivolumab	0.89	3.82	291	Hepatitis	ATN in the setting of acute liver failure, required CVVH, and expired.
**4**	60 yo WM	Stage 3	pembrolizumab	0.84	1.43	175		Clinically adjudicated ICI-associated AKI improved with holding ICI
**5**	70 yo WM	Stage 3	nivolumab	0.77	1.63	280		Clinically adjudicated ICI-associated AKI improved with steroids and holding ICI
**6**	70 yo WM	Stage 3	pembrolizumab	0.81	1.48	203		Clinically adjudicated ICI-associated AKI improved with holding ICI and discontinuing PPI
**7**	80 yo WM	Stage 3	pembrolizumab	1.23	2.57	135		ATN occurring after cardiac surgery
**8**	80 yo WF	Stage 3	pembrolizumab	0.61	1.02	125		Obstructive AKI improved after ureteral stent placement
**9**	65 yo WM	Stage 4	nivolumab	0.99	3.38	119		Clinically adjudicated ICI-associated AKI improved with steroids and holding ICI
**10**	75 yo WF	Stage 4	pembrolizumab	0.77	2.13	220		Pre-renal AKI from diuresis in the setting of severe aortic stenosis
**11**	55 yo WF	Stage 4	pembrolizumab	0.6	1	146	Thyroiditis	Pre-renal AKI improved with supportive therapy
**12**	70 yo WM	Stage 4	nivolumab	1.29	4.11	225		Contrast-induced nephropathy
**13**	85 yo WM	Stage 4	pembrolizumab	1.39	3.18	158		Clinically adjudicated ICI-associated AKI improved withholding ICI and discontinuing PPI
**14**	60 yo WF	Stage 4	pembrolizumab	0.77	1.5	128	Hepatitis	Pre-renal AKI improved with intravenous fluids
**15**	80 yo WF	Stage 4	nivolumab	0.78	1.4	304		Pre-renal AKI improved with intravenous fluids
**16**	80 yo WM	Stage 4	pembrolizumab	0.71	1.44	22		ATN due to hydrochlorothiazide and lisinopril use

**17**	80 yo WM	Stage 4	pembrolizumab	0.71	2.08	337		ATN caused by septic shock
**18**	50 yo WM	Stage 4	nivolumab	0.93	3.3	351		Clinically adjudicated ICI-associated AKI improved with discontinuation of Ciprofloxacin and holding ICI
**19**	55 yo WM	Stage 4	pembrolizumab	0.42	1.04	22		Pre-renal AKI improved spontaneously
**20**	85 yo WM	Stage 4	pembrolizumab	1.03	2.55	279		Clinically adjudicated ICI-associated AKI improved with steroids
**21**	60 yo WF	Stage 4	pembrolizumab	0.77	3.25	152		Pre-renal AKI improved with intravenous fluids
**22**	75 yo WM	Stage 4	pembrolizumab	1.1	2.17	24		ATN in the setting of poor oral intake and failure to thrive, transitioned to hospice and expired
**23**	70 yo WF	Stage 4	pembrolizumab	0.51	0.91	146		Pre-renal AKI improved spontaneously
**24**	70 yo WM	Stage 4	nivolumab	0.87	1.92	111		Clinically adjudicated ICI-associated AKI improved with holding ICI and discontinuing PPI
**25**	85 yo WF	Stage 4	pembrolizumab	0.91	1.61	34		ATN in the setting of MRSA empyema
**26**	50 yo WF	Stage 4	pembrolizumab	0.79	1.52	33		ATN in the setting of acute liver failure which led to death
**27**	60 yo WM	Stage 4	pembrolizumab	0.52	0.93	172		Pre-renal AKI improved spontaneously
**28**	75 yo WM	Stage 4	pembrolizumab	1.36	3.13	122		Pre-renal AKI from diuresis in the setting of congestive heart failure
**29**	35 yo WM	Stage 4	nivolumab	0.37	1.6	1		ATN in the setting of worsening malignant ascites and sepsis, led to death
**30**	60 yo WM	Stage 4	Ipi/nivo	0.72	1.55	105		ATN in the setting of worsening malignant ascites. Transitioned to hospice and expired
**31**	75 yo WM	Stage 4	Ipi/nivo	0.95	1.96	77		Pre-renal AKI improved with intravenous fluids
**32**	55 yo WF	Stage 4	Ipi/nivo	0.54	0.97	70	Colitis	Clinically adjudicated ICI-associated AKI presented with concurrent ICI-colitis, improved with steroids and holding ICI
**33**	70 yo WF	Stage 4	Ipi/nivo	0.43	0.82	77		Pre-renal AKI improved spontaneously
**34**	55 yo WM	Stage 4	Ipi/nivo	1.39	4.25	19		Contrast-induced nephropathy

**35**	65 yo WM	Stage 4	Ipi/nivo	0.75	3.26	29		Clinically adjudicated ICI-associated AKI improved with steroids and holding ICI
**36**	70 yo WF	Stage 4	Ipi/nivo	0.5	1.41	78		ATN in the setting of worsening malignant ascites, transitioned to hospice and deceased
**37**	40 yo WF	Stage 4	Ipi/nivo	0.51	0.94	20	Pyrexia, Hepatitis	Pre-renal AKI improved spontaneously
**38**	65 yo WF	Stage 4	Ipi/nivo	0.76	2.79	35	Hepatitis	Clinically adjudicated ICI-associated AKI improved with steroids and holding ICI
**39**	60 yo WM	Stage 4	Ipi/nivo	1.07	2.75	5		ATN in the setting of worsening malignant ascites, failure to thrive. Transitioned to hospice and expired
**40**	65 yo WM	Stage 4	Ipi/nivo	0.94	2.47	86		Clinically adjudicated ICI-associated AKI improved with steroids and holding ICI
**41**	60 yo WF	Stage 4	Ipi/nivo	0.82	2.52	297		Clinically adjudicated ICI-associated AKI improved with steroids and holding ICI
**42**	65 yo WF	Stage 4	Ipi/nivo	0.82	3.1	331		ATN in the setting of shock and contrast
**43**	60 yo WM	Stage 4	Ipi/nivo	0.89	1.69	75	Hepatitis	Obstructive AKI resolved after PCN placement
**44**	55 yo WF	Stage 4	Ipi/nivo	0.58	2.29	161		ATN in the setting of worsening malignant ascites, transitioned to hospice and expired
**45**	65 yo WF	Stage 4	Ipi/nivo	0.76	2.37	63		Biopsy-confirmed severe AIN improved with steroids and holding ICI
**46**	60 yo WM	Stage 4	Ipi/nivo	0.83	1.4	275		Clinical adjudicated ICI-associated AKI partially improved with holding ICI
**47**	50 yo WM	Stage 4	Ipi/nivo	0.94	2.43	91	Hypophysitis	Biopsy-confirmed AIN, improved with steroids and holding ICI
**48**	50 yo WM	Stage 4	Ipi/nivo	1.14	1.93	70		Pre-renal AKI improved with intravenous fluids
**49**	70 yo WM	Stage 4	Ipi/nivo	0.88	1.44	128		Clinically adjudicated ICI-associated AKI partially improved with holding ICI
**50**	60 yo WM	Stage 4	Ipi/nivo	0.62	0.97	315		Pre-renal AKI improved spontaneously
**51**	65 yo WF	Stage 4	Ipi/nivo	0.61	1.6	13		ATN in the setting of ARDS and shock from high metastatic burden; transitioned to hospice and expired
**52**	55 yo AF	Stage 4	Ipi/nivo	0.71	4.84	195		Clinically adjudicated ICI-associated AKI improved with steroids and holding ICI

**53**	60 yo WM	Stage 4	Ipi/nivo	0.85	1.7	76	Clinical adjudicated ICI-associated AKI with slow response to steroid required a prolonged taper over 2 months
**54**	45 yo WF	Stage 4	Ipi/nivo	0.46	0.81	132	Pre-renal AKI improved spontaneously
**55**	60 yo WM	Stage 4	Ipi/nivo	1.15	2.28	27	Clinically adjudicated ICI-associated AKI improved with steroids and holding ICI
**56**	60 yo WM	Stage 4	Ipi/nivo	0.7	1.71	260	Pre-renal AKI from diarrhea improved with intravenous fluids

Table S3. Case summary of patients who developed sustained AKI within 1-year of ICI initiation. If a patient developed more than one instance of sustained AKI during follow-up, time interval to first AKI episode was documented. Concurrent irAE (defined by non-kidney irAE diagnosed within 14 days of ICI-associated AKI) was present in 4 out of 22 patients (18%) with ICI-associated AKI in our cohort. Among the 9 patients with sustained AKI who had concurrent irAEs: 4 were adjudicated to have ICI-associated AKI, and the other 5 cases were deemed to have hemodynamic cause of AKI. Ages of the patients have been rounded to the nearest 5-year landmarks to protect confidentiality. Abbreviations: WM= white male, WF= white female, AF= Asian female, yo= years old, ipi/nivo= ipilimumab/nivolumab, PPI=proton pump inhibitor, CVVH= continuous veno-venous hemofiltration, MRSA =methicillin-resistant staphylococcus aureus, PCN= percutaneous nephrostomy

Supplementary Table 4. Univariable and multivariable Fine-Gray model predicting composite CKD outcome among patients surviving more than one year

		CKD		
**Characteristics**	**Univariable analysis**	**P-value**	**Multivariable analysis**	**P-value**
	Hazard ratio [95% CI]		Hazard ratio [95% CI]	
**Every 10 years increase in age**	2.13 [1.76, 2.59]	<0.001		
**Age>65**	6.39 [3.24, 12.60]	<0.001	4.61 [2.10, 10.09]	<0.001
**Female sex**	0.90 [0.54, 1.51]	0.70	1.11 [0.65, 1.91]	0.70
**White race**	0.53 [0.17, 1.66]	0.28	0.33 [0.12, 0.90]	0.03
**Stage 3 melanoma**	1		1	
**Stage 4 melanoma with anti-PD-1 monotherapy**	1.00 [0.58, 1.72]	1.00	0.89 [0.51, 1.55]	0.68
**Stage 4 with anti-CTLA-4/PD-1 combination therapy**	0.55 [0.24,1.25]	0.15	0.71 [0.32, 1.64]	0.42
**every 10ml/min/1.73m^2 decrease in baseline eGFR**	1.36 [1.24,1.49]	<0.001	1.14 [1.02, 1.28]	0.02
**Diabetes**	1.24 [0.67,2.31]	0.49		
**HTN**	2.22 [1.25,3.93]	0.006	1.25 [0.68, 2.28]	0.47
**CAD**	1.49 [0.81,2.75]	0.20		
**ACEi/ARB***	1.86 [1.13,3.07]	0.02		
**PPI**	1.58 [0.96,2.61]	0.07	1.30 [0.78, 2.18]	0.32
**Diuretics***	1.94 [1.17,3.22]	0.01		

Table S4. There were 631(N=227 Stage 3, N=270 Stage 4 with anti-PD-1 monotherapy, N=134 Stage 4 with anti-CTLA-4/PD-1 combination therapy) patients who survived more than 365 days. Fine-Gray model included death after 365 days as a competing risk. Baseline demographics (age, race, sex), group were selected a priori for inclusion in multivariable model and baseline variables with a *P* value<0.1 in the univariable model were also included (every 10ml/min/1.73m^2 decrease in baseline eGFR, HTN, PPI). *ACEi/ARB and diuretics were not included in the multivariable model given collinearity with HTN. Stage 3 melanoma was used as the reference group for consistency with prior AKI models. Composite CKD outcome was defined as new onset eGFR<60ml/min/1.73m^2 at least twice separated by at least 90 days without intervening values above 60 or a sustained 30% decline in eGFR compared to baseline for more than 90 days, whichever happened first. Abbreviations: anti-CTLA-4= anti-cytotoxic T-lymphocyte-associated protein 4, anti-PD-1= anti-programmed

cell death protein 1, eGFR = estimated glomerular filtration rate, HTN= hypertension, CAD= coronary artery disease, ACEI/ARB= angiotensin converting enzyme inhibitor/angiotensin receptor blockade, PPI= proton pump inhibitor. Diuretics includes loop, thiazide, and potassium-sparing diuretics.

Supplementary Table 5. Baseline characteristics of patients who survived more than one year by melanoma stage and ICI treatment regimen

Characteristics	All patients surviving more than 1 year (N=631)	Stage 3 melanoma (N=227)	Stage 4 melanoma with anti-PD-1 monotherapy (N=270)	Stage 4 melanoma with anti-CTLA-4/PD-1 combination therapy (N=134)
**Demographics**				
Age (years, SD)	62.9 (13.5)	61.8 (14.1)	66.1 (12.8)	58.1 (12.1)
Female sex (n, %)	250 (39.6%)	93 (41.0%)	104 (38.5%)	53 (39.6%)
Race (n, %)				
White	614 (97.3%)	218 (96.0%)	265 (98.1%)	131 (97.8%)
Other[1]	17 (2.7%)	9 (4.0%)	5 (1.9%)	3 (2.2%)
**Co-existing conditions-no.(%)**				
Hypertension	355 (56.3%)	126 (55.5%)	167 (61.9%)	62 (46.3%)
Diabetes	102 (16.2%)	36 (15.9%)	49 (18.1%)	17 (12.7%)
Cirrhosis	3 (0.5%)	0 (0%)	2 (0.7%)	1 (0.7%)
CAD	103 (16.3%)	40 (17.6%)	47 (17.4%)	16 (11.9%)
**eGFR at ICI initiation (ml/min, SD)**	87.7 (18.2)	87.3 (18.9)	85.5 (18.1)	92.9 (16.4)
**CKD stage at ICI initiation**				
eGFR ≥90 ml/min/1.73m^2	310 (49.1%)	112 (49.3%)	116 (43.0%)	82 (61.2%)
60-89 ml/min/1.73m^2	271 (43.1%)	96 (42.3%)	129 (47.8%)	47 (35.1%)
45-59 ml/min/1.73m^2	35 (5.5%)	13 (5.7%)	19 (7.0%)	3 (2.2%)
30-44 ml/min/1.73m^2	13 (2.1%)	6 (2.6%)	5 (1.9%)	2 (1.5%)
<30 ml/min/1.73m^2	1 (0.2%)	0 (0.0%)	1 (0.4%)	
**Medication use-no.(%)**				
ACEI/ARB	202 (32.0%)	67 (29.5%)	100 (37.0%)	35 (26.1%)
PPI	217 (34.4%)	60 (26.4%)	115 (42.6%)	42 (31.3%)
Diuretics[2]	177 (28.1%)	48 (21.2%)	100 (37.0%)	29 (21.6%)
**ICI agent-no.(%)**				
Pembrolizumab	319 (50.6%)	118 (52.0%)	201 (74.4%)	-
Nivolumab	178 (28.2%)	109 (48.0%)	69 (25.6%)	-
Combination ipi/nivo	134 (21.2%)	-	-	134 (100%)

Table S5. Abbreviations: ICI=immune checkpoint inhibitor, CAD=coronary artery disease, eGFR = estimated glomerular filtration rate, ACEI/ARB = angiotensin converting enzyme inhibitor/angiotensin receptor blockade, PPI=proton pump inhibitor, anti-CTLA-4=anti-cytotoxic T-lymphocyte-associated protein 4, anti-PD-1=anti-programmed cell death protein 1, ipi/nivo=ipilimumab/nivolumab. 1) Includes Asian, Hispanic, African American (total of 6 patients) and those races were not documented (total of 11 patients). 2) Includes loop, thiazide-like, and potassium-sparing diuretics.

Supplementary Table 6. Univariable and multivariable Cox model evaluating association of AKI with mortality in patients survived more than 6 months

Characteristics	Univariable analysis	P-value	Multivariable analysis	P-value
	Hazards ratio [95% CI]		Hazards ratio [95% CI]	
Age	1.00 [1.00,1.01]	0.27	0.99 [0.98, 1.00]	0.20
Female sex	1.18 [0.94,1.48]	0.14	1.15 [0.92, 1.45]	0.22
White race	1.49 [0.83,2.65]	0.18	1.95 [1.08, 3.52]	0.03
Stage 3 melanoma	1		1	
Stage 4 melanoma with anti-PD-1 monotherapy	2.03 [1.53,2.69]	<0.01	2.05 [1.53, 2.73]	<0.01
Stage 4 melanoma with anti-CTLA-4/PD-1 combination therapy	2.14 [1.55, 2.95]	<0.01	2.20 [1.58, 3.06]	<0.01
No AKI	1		1	
ICI-associated AKI	0.85 [0.35, 2.07]	0.73	0.84 [0.34, 2.05]	0.70
Non ICI-associated AKI	1.58 [1.18, 2.12]	0.02	1.46 [1.08, 1.97]	0.01
Baseline eGFR	0.99 [0.99,1.00]	0.01	0.99 [0.98, 1.00]	<0.01
Cirrhosis	0.72 [0.10, 5.14]	0.74		
Diabetes	1.08 [0.81, 1.44]	0.61		
HTN	0.94 [0.75, 1.17]	0.55		
CAD	1.22 [0.92, 1.62]	0.18		
PPI	1.01 [0.80, 1.27]	0.97		
ACEI/ARB	1.02 [0.81, 1.29]	0.86		
Diuretics	1.32 [1.04, 1.67]	0.02	1.12 [0.87, 1.44]	0.37

Table S6. Univariable and multivariable Cox model evaluating association of ICI-associated AKI and non ICI-associated AKI within 6 months of ICI initiation with mortality in patients survived more than 6 months (N=726). ICI-associated AKI was diagnosed either by kidney biopsy or clinical criteria (see method section for further details). Non ICI-associated AKI includes sustained AKI episodes that were adjudicated to be non ICI-related causes (eg. hemodynamic, obstructive) and all non-sustained AKI episodes (presumed to be hemodynamic AKIs given their transient nature). Abbreviations: anti-CTLA-4= anti-cytotoxic T-lymphocyte-associated protein 4, anti-PD-1= anti-programmed cell death protein 1, eGFR = estimated glomerular filtration rate, HTN= hypertension, CAD= coronary artery disease, ACEI/ARB= angiotensin converting enzyme inhibitor/angiotensin receptor blockade, PPI= proton pump inhibitor. Diuretics includes loop, thiazide, and potassium-sparing diuretics.

Supplementary Figure 1. Patient Flow

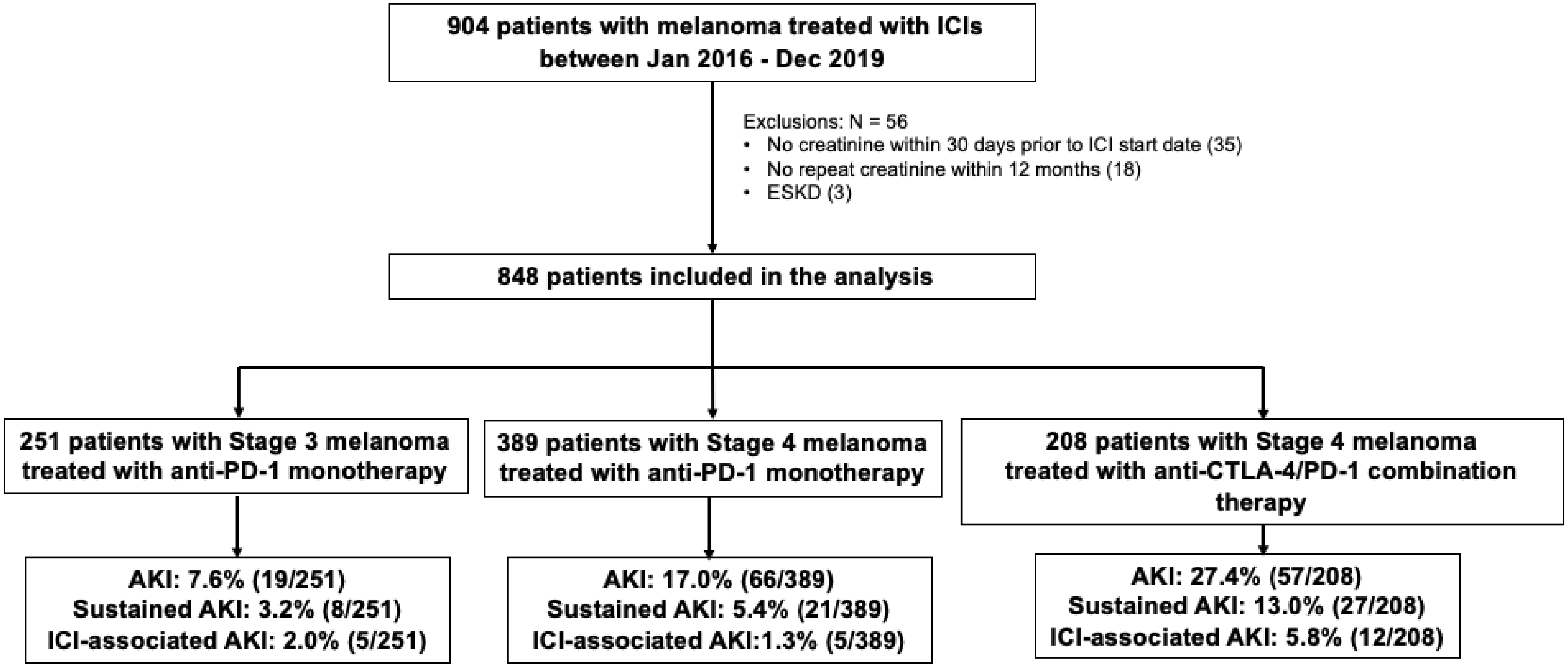

Figure S1. Patient flow. The 848 included patients were categorized into 3 groups based on cancer stage and ICI treatment regimen. Incidence of AKI, sustained AKI and ICI-associated AKI within 1-year of ICI initiation were evaluated between three groups. Abbreviations: ICI= immune checkpoint inhibitor, ESKD= end stage kidney disease, anti-CTLA-4 = anti-cytotoxic T-lymphocyte associated protein 4, anti-PD-1 = anti-programmed cell death protein 1.

Supplementary Figure 2. Cumulative incidence curve for composite chronic kidney disease outcome by melanoma stage and ICI treatment regimen among patients surviving more than one year

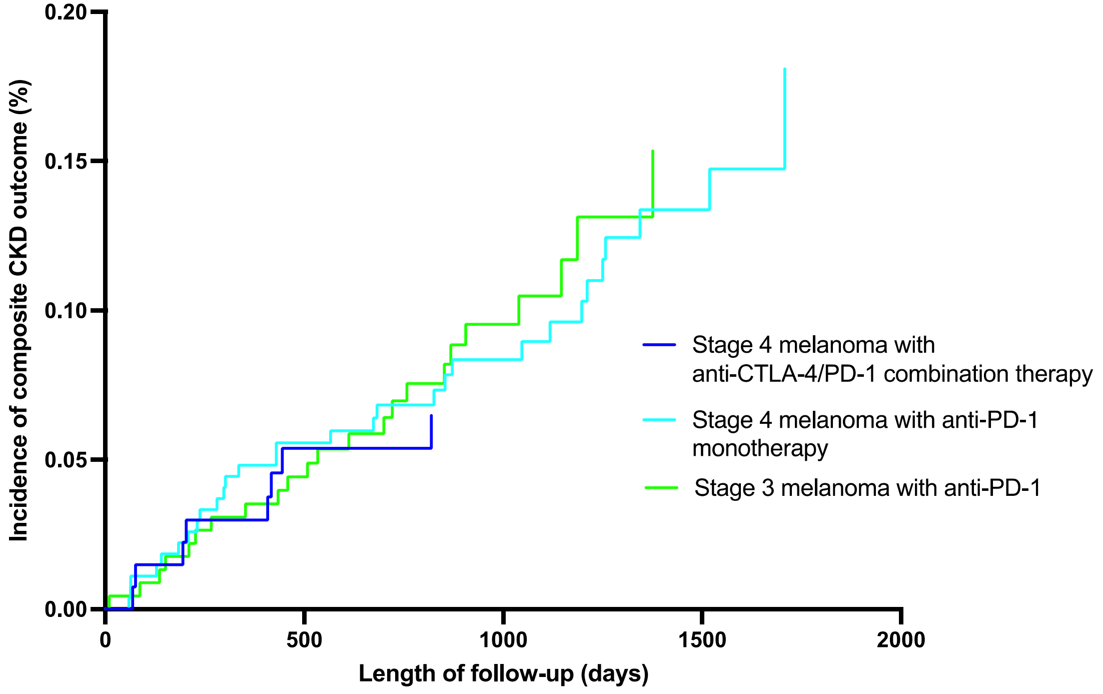

Figure S2. Cumulative incidence curve for composite chronic kidney disease outcome by melanoma stage and ICI treatment regimen among patients surviving more than one year. There was no statistically significant difference in cumulative incidence function (CIF) among the three groups. The CIF curve for Stage 4 melanoma with anti-CTLA-4/PD-1 combination therapy is truncated at day 819 as no patients in that group developed composite CKD outcome afterwards, in the setting of high overriding risk of mortality in that group (21 out of the 62 who survived beyond 2 years died in Stage 4 melanoma with anti-CTLA-4/PD-1 combination therapy [34%], compared to 28% in Stage 4 melanoma with anti-PD-1 monotherapy, and 17% in Stage 3 melanoma with anti-PD-1 monotherapy, respectively). All three groups had equivalent length of follow-up: median 888 days [568, 1123] in Stage 3 melanoma with anti-PD-1 monotherapy, 813 days [547, 1224] in Stage 4 melanoma with anti-PD-1 monotherapy, and 703 days [449, 1200] in Stage 4 melanoma with anti-CTLA-4/PD-1 combination therapy group.

Supplementary references

S1. Hodi FS, O'Day SJ, McDermott DF, et al. Improved Survival with Ipilimumab in Patients with Metastatic Melanoma. New England Journal of Medicine. 2010;363(8):711-723.

S2. Robert C, Schachter J, Long GV, et al. Pembrolizumab versus Ipilimumab in Advanced Melanoma. New England Journal of Medicine. 2015;372(26):2521-2532.

S3. Eggermont AMM, Blank CU, Mandala M, et al. Adjuvant Pembrolizumab versus Placebo in Resected Stage III Melanoma. New England Journal of Medicine. 2018;378(19):1789-1801.

S4. Weber J, Mandala M, Del Vecchio M, et al. Adjuvant Nivolumab versus Ipilimumab in Resected Stage III or IV Melanoma. New England Journal of Medicine. 2017;377(19):1824-1835.

S5. Espi M, Teuma C, Novel-Catin E, et al. Renal adverse effects of immune checkpoints inhibitors in clinical practice: ImmuNoTox study. Eur J Cancer. 2021;147:29-39.

S6. Shirali AC, Perazella MA, Gettinger S. Association of Acute Interstitial Nephritis With Programmed Cell Death 1 Inhibitor Therapy in Lung Cancer Patients. Am J Kidney Dis. 2016;68(2):287-291.

S7. Meraz-Muñoz A, Amir E, Ng P, et al. Acute kidney injury associated with immune checkpoint inhibitor therapy: incidence, risk factors and outcomes. J Immunother Cancer. 2020;8(1).

S8. Isik B, Alexander MP, Manohar S, et al. Biomarkers, Clinical Features, and Rechallenge for Immune Checkpoint Inhibitor Renal Immune-Related Adverse Events. Kidney Int Rep. 2021;6(4):1022-1031.

S9. Shimamura Y, Watanabe S, Maeda T, Abe K, Ogawa Y, Takizawa H. Incidence and risk factors of acute kidney injury, and its effect on mortality among Japanese patients receiving immune check point inhibitors: a single-center observational study. Clin Exp Nephrol. 2021;25(5):479-487.

S10. Oleas D, Bolufer M, Agraz I, et al. Acute interstitial nephritis associated with immune checkpoint inhibitors: a single-centre experience. Clin Kidney J. 2021;14(5):1364-1370.

S11. Seethapathy H, Zhao S, Strohbehn IA, et al. Incidence and Clinical Features of Immune-Related Acute Kidney Injury in Patients Receiving Programmed Cell Death Ligand-1 Inhibitors. Kidney Int Rep. 2020;5(10):1700-1705

S12. Baker ML, Yamamoto Y, Perazella MA, et al. Mortality after acute kidney injury and acute interstitial nephritis in patients prescribed immune checkpoint inhibitor therapy. J Immunother Cancer. 2022;10(3).

S13. Choueiri TK, Tomczak P, Park SH, et al. Adjuvant Pembrolizumab after Nephrectomy in Renal-Cell Carcinoma. New England Journal of Medicine. 2021;385(8):683-694.

S14. Schmid P, Cortes J, Pusztai L, et al. Pembrolizumab for Early Triple-Negative Breast Cancer. New England Journal of Medicine. 2020;382(9):810-821.

S15. Kelly RJ, Ajani JA, Kuzdzal J, et al. Adjuvant Nivolumab in Resected Esophageal or Gastroesophageal Junction Cancer. New England Journal of Medicine. 2021;384(13):1191-1203.

S16. Felip E, Altorki N, Zhou C, et al. Adjuvant atezolizumab after adjuvant chemotherapy in resected stage IB-IIIA non-small-cell lung cancer (IMpower010): a randomised, multicentre, open-label, phase 3 trial. Lancet. 2021;398(10308):1344-1357.

S17. McMahon BA, Rosner MH. GFR Measurement and Chemotherapy Dosing in Patients with Kidney Disease and Cancer. Kidney360. 2020;1(2):141-150.
S18. Costa ESVT, Gil LA, Jr., Inker LA, et al. A prospective cross-sectional study estimated glomerular filtration rate from creatinine and cystatin C in adults with solid tumors. Kidney Int. 2022;101(3):607-614.
S19. Keung EZ, Gershenwald JE. The eighth edition American Joint Committee on Cancer (AJCC) melanoma staging system: implications for melanoma treatment and care. Expert Rev Anticancer Ther. 2018;18(8):775-784.
S20. Brahmer JR, Abu-Sbeih H, Ascierto PA, et al. Society for Immunotherapy of Cancer (SITC) clinical practice guideline on immune checkpoint inhibitor-related adverse events. Journal for ImmunoTherapy of Cancer. 2021;9(6):e002435.
S21. Inker LA, Eneanya ND, Coresh J, et al. New Creatinine- and Cystatin C–Based Equations to Estimate GFR without Race. New England Journal of Medicine. 2021;385(19):1737-1749.
